# Editorial: Vitamin D in Neurological Diseases: From Pathophysiology to Therapy

**DOI:** 10.3389/fneur.2021.614900

**Published:** 2021-03-09

**Authors:** Joost Smolders, Amie Hiller, William Camu

**Affiliations:** ^1^Departments of Neurology and Immunology, MS Center ErasMS, Erasmus Medical Center, Rotterdam, Netherlands; ^2^Department of Neuroimmunology, Netherlands Institute for Neuroscience, Amsterdam, Netherlands; ^3^Oregon Health & Science University Neurology Clinic, Portland, OR, United States; ^4^VA Portland Health Care System, Portland, OR, United States; ^5^ALS Center, CHU and Univ Montpellier, Montpellier, France

**Keywords:** vitamin D, biomarker, Parkinson's disease, ALS, multiple sclerosis

Despite decades of studies associating a low vitamin D status with detrimental disease outcomes that cover the whole landscape of neurological diseases, the role of vitamin D in these disorders is a controversial issue. With this review topic, we aimed to bring together pre-clinical, epidemiological and clinical studies addressing the role of vitamin D in neurological diseases.

In this Research Topic, three papers were review articles addressing the role of vitamin D in specific neurological diseases. Miclea et al. review work on vitamin D and multiple sclerosis (MS), Fullard and Duda review the association of vitamin D with Parkinson's disease, and Lanznaster et al. review a role of vitamin D supplements in Amyotrophic Lateral Sclerosis (ALS). Interestingly, all three reviews draw focus to association studies on vitamin D and disease incidence and outcome, and on clinical supplementation studies on improvement of disease outcomes. The conclusions drawn by the authors of these papers show striking similarities. The association of low 25-hydroxyvitamin D [25(OH)D] levels with an increased risk of these diseases, and with adverse outcomes on relevant disease endpoints, has been frequently reported and reproduced in all these diseases. Confounding is a huge issue in analyzing the date in all three disease - whether these low levels are causes of or consequence of underlying disease processes and associated disability, remains uncertain from the data reviewed by the authors. Vitamin D supplementation studies targeting these endpoints can be regarded as the most straightforward approach to bridge this knowledge gap. However, inconsistent results of clinical trials are reported, with substantial heterogeneity in trial-design, trial-duration, intervention strategies and (size of) trial populations. The abundance of negative trials brings up the question if normal vitamin D levels may be more a reflection of underlying health status. Trials have been mostly negative on primary endpoints, but showed signals of benefit in secondary endpoints. Rigorous clinical studies have been identified as a general need to provide clarity about a possible role of vitamin D supplements as a treatment of neurological diseases.

In addition to these thorough reviews of current status and needs of the field, other studies introducing new data and concepts were presented with the most not surprisingly in the field of MS, which has been at the forefront of examining a possible role for vitamin D.

Associations of low 25(OH)D levels with disease incidence and outcome were reported by three papers in this Research Topic. Chouët et al. report lower 25(OH)D levels in a cross-sectional study among *N* = 60 geriatric inpatients with delirium compared to *N* = 180 matched controls. At the threshold value of 30 nmol/L, 25(OH)D levels were most clearly associated with geriatric delirium, with also a linear association being present. Severe vitamin D deficiency had been associated with a more severe disease and earlier death in ALS patients, but those results remained in dispute (1). In the present issue Lanznaster et al. with a meta analysis of the literature underline the importance of taking into consideration confounders. As a kind of answer, Juntas-Morales et al. report in a prospective cohort of *N* = 127 patients with ALS that lower 25(OH)D levels at study entrance associated with a higher ALS severity score of worsening. These levels were not associated with overall survival. Importantly, associations were corrected for confounders in multivariate models and 25(OH)D levels were the only parameter associated with ALS severity. In several neurological diseases, circulating and CSF neurofilament light-chain (NfL) levels are under investigation as a biomarker of axonal damage (2). The association of NfL-levels with 25(OH)D levels was assessed in a prospectively followed clinical trial cohort of *N* = 85 newly-diagnosed relapsing remitting MS patients supplemented with omega 3 fatty acids by Røsjø et al. No associations between natural variation in NfL and 25(OH)D levels were observed. These results confirm these absence of a large effect of vitamin D supplements on generally relatively low NfL levels reported in several small clinical trials in MS (5, 7, 9).

Most experimental studies on vitamin D and multiple sclerosis addressed effects of vitamin D on immune cells. Lee et al. report results of experimental studies *in vitro* and in the experimental autoimmune encephalitis (EAE) model of neuroinflammation. They report that exposure of neurons to vitamin D promoted an anti-inflammatory state in microglia, which suggests an indirect effect of vitamin D on CNS immune cell behavior, by impacting resident cells as neurons. This effect may be taking place early in life, as suggested by findings from the neuron-specific partial vitamin D receptor knockout animal model. Interestingly, environmental effects on the risk of MS have also been attributed to early like exposure in early life in several epidemiological studies (4).

Regarding dosing of vitamin D_3_ supplements in clinical trials Häusler et al. express a note of caution for deleterious effects of very highly dosed vitamin D by disturbing intracellular calcium signaling. They review, among other papers, their earlier work, showing high-dose vitamin D supplementation in the EAE animal-model of neuroinflammation to coincide with hypercalcemia and with a clinically detrimental and immunologically more inflammatory disease course (6). Caution for hypercalcemia in high-dose supplementation trials is advised based on these data and hypercalcemia as a known hallmark of vitamin D toxicity, but has not been encountered in several clinical trials in MS thus far (8). An added value for neurological disease outcomes of targeting very high 25(OH)D levels instead of preventing the poorest vitamin D statuses remains, however, unsupported by literature and may have even detrimental effects. Data from looking at elderly fallers has clearly shown that very high doses can increase the risk of falling and many suggest against mega dosing of vitamin D (3).

Altogether, these studies provided additional insights in the role of vitamin D in neurological diseases, yet also provided additional insights in critical gaps in our knowledge which need to be bridged. Remarkably, these challenges are fairly consistent throughout the range of different neurological diseases associated with a poor vitamin D status. The studies in this collection emphasized the need for rigorous clinical studies on vitamin D supplementation targeting disease relevant endpoints in relevant cohorts of patients and making sure that potential confounders are included as part of the study design. This is ultimately the most straightforward way to answer the question whether there is a benefit in taking vitamin D supplements for (subgroups of) patients.

## Author Contributions

All authors supervised the whole content of editorial.

## Conflict of Interest

JS received speaker and/or consultancy fee from Biogen, Merck, Novartis, and Sanofi-Genyzme. The remaining authors declare that the research was conducted in the absence of any commercial or financial relationships that could be construed as a potential conflict of interest.
